# Immunization against *Leishmania major* Infection Using LACK- and IL-12-Expressing *Lactococcus lactis* Induces Delay in Footpad Swelling

**DOI:** 10.1371/journal.pone.0030945

**Published:** 2012-02-10

**Authors:** Felix Hugentobler, Karen K. Yam, Joshua Gillard, Raya Mahbuba, Martin Olivier, Benoit Cousineau

**Affiliations:** 1 Department of Microbiology and Immunology, McGill University, Montréal, Québec, Canada; 2 Centre for the Study of Host Resistance, Research Institute of the McGill University Health Centre, Montréal, Québec, Canada; 3 Member of the Centre for Host-Parasite Interaction (CHPI), Ste. Anne de Bellevue, Québec, Canada; Technion-Israel Institute of Technology Haifa 32000 Israel., Israel

## Abstract

**Background:**

*Leishmania* is a mammalian parasite affecting over 12 million individuals worldwide. Current treatments are expensive, cause severe side effects, and emerging drug resistance has been reported. Vaccination is the most cost-effective means to control infectious disease but currently there is no vaccine available against Leishmaniasis. *Lactococcus lactis* is a non-pathogenic, non-colonizing Gram-positive lactic acid bacterium commonly used in the dairy industry. Recently, *L. lactis* was used to express biologically active molecules including vaccine antigens and cytokines.

**Methodology/Principal findings:**

We report the generation of *L. lactis* strains expressing the protective *Leishmania* antigen, LACK, in the cytoplasm, secreted or anchored to the bacterial cell wall. *L. lactis* was also engineered to secrete biologically active single chain mouse IL-12. Subcutaneous immunization with live *L. lactis* expressing LACK anchored to the cell wall and *L. lactis* secreting IL-12 significantly delayed footpad swelling in *Leishmania major* infected BALB/c mice. The delay in footpad swelling correlated with a significant reduction of parasite burden in immunized animals compared to control groups. Immunization with these two *L. lactis* strains induced antigen-specific multifunctional T_H_1 CD4^+^ and CD8^+^ T cells and a systemic LACK-specific T_H_1 immune response. Further, protection in immunized animals correlated with a *Leishmania*-specific T_H_1 immune response post-challenge. *L. lactis* secreting mouse IL-12 was essential for directing immune responses to LACK towards a protective T_H_1 response.

**Conclusions/Significance:**

This report demonstrates the use of *L. lactis* as a live vaccine against *L. major* infection in BALB/c mice. The strains generated in this study provide the basis for the development of an inexpensive and safe vaccine against the human parasite *Leishmania*.

## Introduction

Leishmaniasis, caused by the protozoan parasites of the genus *Leishmania*, affects over 12 million individuals worldwide, 1.5–2.0 million of whom develop symptomatic disease every year [1]. The parasite is spread by the sand fly vector and causes a spectrum of diseases depending on the parasite species and the host immune status. Clinical manifestations of the human disease range from self-limiting cutaneous leishmaniasis, disfiguring mucocutaneous leishmaniasis, to fatal visceral leishmaniasis. Current treatments use toxic pentavalent antimonial compounds. These treatments are laborious and expensive, cause severe side effects and emerging drug resistance has been reported [2]. Therefore, an effective *Leishmania* vaccine would be desirable. Leishmanisation using live *Leishmania major* parasites generates long-lasting protective immunity against further infection, indicating that the development of a *Leishmania* vaccine is feasible [1].

Immunity against *L. major* infection is well characterized in the mouse model and depends mainly on T_H_1 immune responses mediated by CD4^+^ T cells induced by the essential cytokine IL-12 [3], [4]. However, a role for CD8^+^ T and NK cells has been described in acquired resistance against *Leishmania* [5], [6]. Several protective antigens against *L. major* have been characterized and protection has been reported in animal models after immunization with antigen-encoding DNA vectors or recombinant proteins formulated with T_H_1-inducing adjuvants, such as IL-12 or TLR ligands [7]. One of the best-studied *L. major* antigens is the *Leishmania* homologue of activated C kinase (LACK). It was first identified as a T cell epitope from soluble *Leishmania* antigens (SLA) that conferred protection against *L. major* challenge [8]. This antigen has since been found to be highly conserved among *Leishmania* strains, although its function is not clear. It was demonstrated that LACK is crucial for the viability of the parasite as well as for host infection and parasite reproduction in infected macrophages [9]. Furthermore, it was suggested that LACK plays a role in DNA replication and RNA synthesis [10]. LACK has been shown to be protective against *L. major* challenge in mice as a recombinant protein vaccine administered with recombinant IL-12 as an adjuvant [8]. It was also shown to be effective as DNA or recombinant Vaccinia virus vaccine in different studies [11]–[14]. LACK was also tested as a heterologous live bacterial vaccine. Administration of LACK-expressing *Listeria monocytogenes* partially protected mice against *L. major* challenge [15], [16], whereas immunization with LACK-expressing *Salmonella enterica* as part of a DNA prime, live bacteria-boost strategy was found to enhance protection against *L. major* challenge [17].

*Lactococcus lactis* is a lactic acid bacterium commonly used in the dairy industry and is known to be non-pathogenic and non-colonizing [18]. Accordingly, *L. lactis* was given Generally Recognized As Safe (GRAS) status by the U.S. Food and Drug Administration [19]. For over a decade, *L. lactis* has been used as a live bacterial delivery vector [20]. Early studies showed that genetically modified *L. lactis* heterologously expressing fragment C of the tetanus toxin (TTFC) was able to elicit antigen-specific immune responses and protect against disease [21]–[23]. Since then, many studies have demonstrated the use of *L. lactis* to express and deliver antigens and biologically active molecules such as cytokines [24], [25]. In 2006, *L. lactis* expressing human IL-10 was used in a Phase I trial to treat patients with Crohn's disease [26]. The authors demonstrated that high consecutive daily doses of *L. lactis* administered orally were well-tolerated. Recently, a Phase II clinical trial using strains of *L. lactis* secreting human IL-10 was completed and other pre-clinical trials are being prepared using *L. lactis* to treat various diseases [27].

We have previously demonstrated that *L. lactis* elicits an innate inflammatory response and has the ability to modulate DC maturation, which indicates a capacity for adjuvanticity [28]. In addition, we showed that *L. lactis*-based vaccines expressing the A2 antigen from *Leishmania donovani* are a feasible approach in the generation of live vaccines against visceral leishmaniasis in mice [29]. In this study, we generated strains of *L. lactis* expressing the LACK antigen of *Leishmania* at different subcellular localizations (in the cytoplasm, secreted, or anchored to the cell-wall). We also generated a strain of *L. lactis* secreting mouse single chain IL-12. IL-12 is a potent heterodimeric cytokine that induces T_H_1 cells, enhances CTL maturation, promotes NK cell activity and induces IFN-γ production [30]. Furthermore, IL-12 possesses adjuvant properties and plays an essential role in immunity against *L. major* [3]. We demonstrate that subcutaneous immunization with *L. lactis* displaying the LACK antigen on the cell surface in combination with *L. lactis* secreting mouse single-chain IL-12 was able to elicit antigen-specific humoral and cellular immune responses and reduced parasite burden in *L. major* infected BALB/c mice.

## Materials and Methods

### Bacterial strains and growth conditions

*Escherichia coli* strain DH5α was used for DNA cloning. *E. coli* strain BL21 (DE3) was used for expression and purification of His-LACK. *E. coli* strains were grown with shaking in LB broth (Wisent; St. Bruno, QC, Canada) at 37°C. *L. lactis* subsp. *cremoris* strain NZ9000 was grown without shaking in M17 medium (Oxoid; Basingstoke, UK) supplemented with 0.5% glucose (GM17) or, where indicated, in three times the manufacturer's recommended concentration of M17 supplemented with glucose (G3xM17) at 30°C. The NZ9000 strain is a plasmid free derivative of the dairy starter strain NCDO71 that is suitable for use as a live vaccine vector [31]. Antibiotics were added at the following concentrations: ampicillin (Amp) 100 µg/ml; spectinomycin (Spec) 300 µg/ml; chloramphenicol (Cam) 10 µg/ml.

### Plasmids

The pDL278 (pDL) *E. coli*-*L. lactis* shuttle plasmid containing a nisin-inducible promoter (P_nisA_) was used for antigen expression as previously described [29]. The LACK gene was PCR amplified from *L. donovani* 1S-2D genomic DNA (NsiI). The LACK gene was cloned downstream of the P_nisA_ promoter (NsiI) to direct protein expression in the cytoplasm of *L. lactis* (pDL-PnisA-cytoLACK). DNA sequencing confirmed that the cloned gene matches the sequence of LACK from *L. donovani* [32] and *L. major* [8]. As previously described, the Usp45 secretion signal was PCR amplified from pCWA:E7 (kindly provided by Dr. Langella) [33], [34] and cloned between the promoter and the LACK gene of the cytoplasmic construct (NsiI) to direct protein expression to the culture medium (pDL-PnisA-secLACK). To add the cell-wall anchoring (cwa) domain, a restriction site was first introduced at the 3′-end of the LACK gene. A fragment including the P_nisA_ promoter, the secretion signal and the LACK gene was PCR amplified to add a NheI site before the stop codon and was cloned into pDL. The cwa domain was PCR amplified from pCWA:E7 and was cloned downstream of the LACK gene (NheI). The secretion signal (N-terminus) and the cwa domain (C-terminus) directs the LACK protein to be anchored to the cell-wall (pDL-PnisA-cwaLACK).

To create a N-terminal His-tagged version of LACK for protein purification from *E. coli*, the LACK gene was PCR amplified from *L. donovani* 1S-2D genomic DNA (NdeI/BamHI) and cloned into pET-16b (Novagene; Darmstadt, Germany) (NdeI/BamHI).

To generate a *L. lactis* strain secreting mouse IL-12, a codon optimized version of the single chain mouse IL-12 gene [35] was engineered (NsiI/NheI) (scIL-12opt, GenScript; Piscataway, NJ). The optimized gene was introduced into pBluescript II SK- (pBS) (SalI/XbaI). The nisin-inducible promoter (P_nisA_) was PCR amplified from the pSEC-scIL-12 template kindly provided by Dr. Bermúdez-Humarán [35] and was cloned directly upstream of scIL-12opt (SalI/NsiI). The secretion signal was PCR amplified from pCWA:E7 and then introduced between the promoter and the gene (NsiI). The expression cassette was excised from pBS (SalI/BamHI) and introduced into the *E. coli*-*L. lactis* shuttle plasmid pLE1 (pLE1-PnisA-secIL-12) [36]. To create a N-terminal His-tagged version of scIL-12opt for protein purification from *E. coli*, the optimized scIL-12 gene was excised from pLE1-PnisA-secIL-12 (NsiI), blunted with T4 DNA Polymerase, and digested with BamHI. The fragment was cloned into pET-16b (Novagene), which had been previously digested with XhoI, blunted, and then digested with BamHI. All plasmid constructs were confirmed by DNA sequencing. Primers used for PCR amplifications are shown in Table 1.

**Table 1:** Bacterial strains and primers used.

Strain name	Description	Source
*L. lactis*/vector	NZ9000/pLE1	Mills *et al.* [36]
*L. lactis*/cytoLACK	NZ9000/pDL-PnisA-LACK	This study
*L. lactis*/secLACK	NZ9000/pDL-PnisA-sec-LACK	This study
*L. lactis*/cwaLACK	NZ9000/pDL-PnisA-sec-LACK-cwa	This study
*L. lactis*/secIL-12	NZ9000/pLE1-PnisA-sec-scIL-12opt	This study
*L. lactis*/secIL-12 wt	NZ9000/pSEC-PnisA-sec-scIL-12 wt	Bermúdez-Humarán *et al.* [35]

Primer-pair name	Description	Sequence (F, forward; R, reverse)
LACKpDL	PCR-amplify LACK gene for cloning into pDL278	F: 5′-CCA ATG CAT AAC TAC GAG GGT CAC CTG AAG-3′R: 5′-CCA ATG CAT TTA CTC GGC GTC GGA GAT GG-3′
Sec signal	PCR-amplify Usp45 secretion signal	F: 5′-CCA ATG CAT AAA AAA AAG ATT ATC TCA GCT AT-3′R: 5′-CCA ATG CAT ATC TGA GTT TGT GTC AGC G-3′
LACK3′NheI	PCR-amplify a fragment containing the nisin promoter, secretion signal and LACK gene to add 3′ NheI site (cloning site for cwa domain)	F: 5′-CCG GAA TTC AGT CGA CCT AGT CTT ATA ACT ATA CTG ACA ATA G-3′R: 5′-AGC TGG CTA GCT CGG CGT CG GAGA TGG ACC A-3′
CWA	PCR-amplify cell-wall anchoring domain from M6 protein of *Streptococcus pyogenes*	F: 5′-CCA ATG CAT CGC TAG CTT TAG AAG AAG CAA ACA-3′R: 5′-TGA GTG CTA GCT TAG TTT TCT TCT TTG CG-3′
LACKpET	PCR-amplify LACK gene for cloning into pET-16b	F: 5′-GGG AAT TCC ATA TGA ACT ACG AGG GTC ACC TG-3′R: 5′-CGC GGA TCC TTA CTC GGC GTC GGA GAT GG-3′
IL-12 PnisA	PCR-amplify PnisA promoter for cloning into pBS-scIL-12opt	F: 5′-CAC CAG TAC TAG TCG ACC TAG TCT TAT AAC TAT ACT GAC AAT AGA AAC ATT-3′R: 5′-TGC GGA ATT CAT GCA TTT TGA GTG CCT CCT TAT AAT TTA T-3′

### Protein expression and Western blotting

Saturated overnight cultures of *L. lactis* were diluted (∼1/20) in fresh media with antibiotics and grown until OD_600_ = 0.4–0.5. Protein expression was induced with the addition of nisin to a final concentration of 10 ng/ml and bacteria were grown for an additional 3 hours. 20 ml of bacterial culture was centrifuged at 4,300× g for 10 min at 4°C; the supernatant was directly used for Western blotting and the cell pellet was used for total cell protein extraction. Bacterial cells were resuspended in 100 µl TES-LMR (10 mM Tris-HCl, 1 mM EDTA (TE) buffer, pH 8.0, with 25% sucrose, 1 mg/ml lysozyme, 50 U/ml mutanolysin, and 0.1 mg/ml RNaseA) and incubated at 37°C for 1 hour. The cells were lysed by the addition of 100 µl of TE+4% SDS and then incubated in boiling water for 5 min. Protein preparations were resolved on 12% SDS-PAGE and transferred onto PVDF membrane (Immobilon-P, Millipore; Billerica, MA) for Western blotting.

Membranes were blotted with polyclonal anti-LACK antibodies kindly provided by Dr. Antoine [37], diluted 1∶30,000 in PBS-1% Tween-20 (PBS-T, Fisher; Ottawa, ON) containing 5% non-fat dried milk. Anti-rabbit antibodies conjugated to horse radish peroxidase (HRP, Sigma-Aldrich, Oakville, ON) were used as the secondary antibody at a dilution of 1∶10,000 in PBS-T+5% milk. To confirm the localization of LACK expressed from different plasmid constructs in *L. lactis*, a whole cell ELISA protocol was employed as previously described [29]. To detect mouse IL-12, membranes were blotted with anti-mouse IL-12 neutralizing antibody (R&D Systems, Minneapolis, MN) 1∶10,000 in PBS-T+5% milk. Anti-goat-HRP (Sigma) was used as secondary antibody at a dilution of 1∶10,000 in PBS-T+5% milk. Proteins were detected using Immobilon Western Chemiluminescent HRP Substrate (Millipore) and visualized using the VersaDoc Molecular Imager (Bio-Rad; Hercules, CA) with QuantityOne software (Bio-Rad).

To determine the levels of single chain IL-12 secreted by *L. lactis*, bacteria were grown in G3xM17 and induced with nisin as described above. Proteins in bacterial supernatant were then concentrated using 50 k Amicon Ultra Centrifugal Filter Units (Millipore) and the concentration of mouse IL-12p70 was determined by ELISA (eBioscience, San Diego, CA).

His-tagged LACK was purified from *E. coli* as previously described [29]. The purified LACK protein was dialysed in PBS (3,5 MWCO, 3–12 ml, Thermo Scientific; Waltham, MA) according to manufacturer's protocol before usage in *ex vivo* stimulation assays.

### *In vitro* IFN-γ bioassay

Spleens from BALB/c mice were mechanically homogenized and erythrocytes were lysed using ACK lysis buffer. Cells were plated in 24-well plates at 2×10^6^ cells/ml in RPMI (Wisent) supplemented with 10% FBS (Wisent), 1 mM penicillin-streptomycin and 0.5 mM β-ME. Cells were stimulated with 100, 50, or 25 pg/ml of rIL-12 (R&D systems) or with concentrated bacterial supernatants (*L. lactis*/secIL-12, *L. lactis*/vector). The supernatant from *L. lactis*/secIL-12 was adjusted to provide ∼50 pg/ml of IL-12 per sample as determined by ELISA. Cells were co-stimulated with anti-CD3 and anti-CD28 (0.5 µg/ml, 2.5 µg/ml, provided by Dr. Fournier, McGill University). Anti-mIL-12 neutralizing antibody (1 µg/ml, R&D Systems) was added to some samples as indicated. Cells were incubated at 37°C under 5% CO_2_ for 24 hours and IFN-γ concentration in the supernatant was determined by ELISA (eBioscience).

### Preparation of live bacteria for immunization

Saturated overnight cultures of *L. lactis* were diluted (∼1/20) in fresh media with antibiotics and grown until OD_600_ = 0.4–0.5. Protein expression was induced with the addition of nisin to a final concentration of 10 ng/ml and was grown for an additional 3 hours. *L. lactis* cells were collected by centrifugation, washed twice in PBS, and resuspended in PBS+25% glycerol at 1/25th of the starting culture volume. Aliquots of induced bacteria were stored at −80°C. Prior to immunization, frozen aliquots were thawed on ice, cells were spun down at 3,000 g at 4°C for 10 min and resuspended in sterile PBS to obtain the desired concentration. Serial dilutions and colony forming units (CFU) counts were performed on each batch of nisin-induced bacteria to determine the dilution required to obtain ∼10^10^ CFU/ml. For each batch of live *L. lactis* inocula prepared, protein expression was confirmed by immunoblotting. Amounts of expressed protein per inoculum (200 µl of bacteria) were determined by Western blot of whole cell protein extracts as described above. Signals were quantified using the “Volume Analysis” function from the QuantityOne software (Bio-Rad) using hisLACK (400 ng) or hisIL-12 (100 ng) purified from *E. coli* as standards.

### Mouse strains

Six to eight week old BALB/c mice were purchased from Charles River Laboratories (Montreal, QC) and maintained in the Duff Medical building animal facility under pathogen-free conditions. All experiments were performed in accordance with guidelines of the Canadian Council on Animal Care, as approved by the Animal Care Committee of McGill University.

### Immunization protocol

LACK and IL-12-expressing *L. lactis* were prepared as described above. Groups of mice were subcutaneously immunized in the back every second week for a total of three immunizations (day 0, day 14, day 28). Mice were immunized with 0.5×10^9^ live bacteria of one of the three strains expressing LACK in combination with 0.5×10^9^ of live *L. lactis* expressing either secIL-12 or carrying the empty vector (total dose of 1×10^9^ live bacteria). As controls, we immunized mice with PBS only, with 1×10^9^ of live *L. lactis*/vector, or with 0.5×10^9^ live *L. lactis*/secIL-12 in combination with 0.5×10^9^ live *L. lactis*/vector. All immunizations were performed in a total volume of 200 µl. Mice were challenged with *Leishmania major* Friedlin V9 infection on day 42.

### Parasite challenge and SLA preparation

*Leishmania major* Friedlin V9 was maintained at 25°C in SDM medium supplemented with 10% FBS (Wisent) as previously described [38]. Mice were challenged with 5×10^6^ late-stationary phase *L. major* promastigotes in 50 µl PBS injected subcutaneously into the right hind footpad two weeks following the last immunization (day 42). Disease progression was monitored at weekly intervals, by measuring the thickness of the infected footpad using a digital vernier calliper and subtracting the thickness of the contralateral uninfected footpad.

Footpad parasite burdens were determined by limiting dilution as previously described [29]. The parasite burden of each footpad was determined from the highest dilution in which viable promastigotes could be grown after 7 days of culture at 25°C in SDM. For some experiments, footpad parasite burdens were determined by PCR-ELISA as previously described [39]. In brief, tissue from infected footpads was homogenized and DNA was isolated using TRIzol reagent (Invitrogen, Carlsbad, CA). DNA concentrations were determined using NanoDrop (Thermo Scientific) and samples were diluted to a final concentration of 10 ng/µl. Parasite DNA for PCR standards was prepared using the proteinase procedure [39]. PCR was performed using digoxigenin-labelled forward and biotin-labelled reverse primers specific for kinetoplast minicircle DNA [39]. Cycling conditions for PCR were as follows; 1 cycle at 94°C for 90 s, followed by 20 cycles of 94°C for 30 s, 53°C for 45 s, 72°C for 60 s and a final extension for 10 min at 72°C using a TGradient thermocycler (Biometra, Göttingen, Germany). ELISA was performed as previously described, except that plates were blocked after coating with PBS+5% FBS for 30 min [39].

To prepare soluble *Leishmania* antigen (SLA), late-stationary phase *L. major* promastigotes were resuspended in sterile PBS to a concentration of ∼10^8^ parasites/ml. Parasites were lysed by five cycles of freeze-thawing, then centrifuged at 4°C for 15 min. The supernatant (SLA) was collected and protein concentration was determined by Bradford assay (BioRad).

### Serum antibody analysis

To determine end-point titers of LACK-specific antibodies, blood was collected from immunized animals from the lateral saphenous vein on day 35, four and ten weeks after parasite challenge. Serum samples were stored at −80°C until analysis. ELISA plates were coated with purified LACK protein (2.5 µg/ml; 50 µl/well) at 4°C overnight. Before and after every subsequent step, wells were washed with PBS+0.5% Tween-20 (PBS-T). Wells were blocked with PBS+2.5% FBS for 1 hour. Two-fold serial dilutions of serum samples were prepared on the ELISA plate at a starting dilution of 1/50 in PBS-FBS and incubated for 2 hours at room temperature. Wells were then incubated with secondary anti-mouse total IgG-HRP antibodies (Sigma) and ABTS ELISA substrate (Sigma), and finally, plates were read at 405 nm in a microplate reader (Bio-Tek; Winooski, VT). The end-point was determined as the highest serum dilution to reach at least the same absorbance reading as the average plus two standard deviations of naïve pre-immune sera (1∶50 dilutions).

To determine the levels of LACK-specific IgG_1_ and IgG_2a_ antibodies, two-fold serial dilutions of individual serum samples were added on ELISA plates coated as above with purified LACK protein. Then, goat anti-mouse IgG_1_ or IgG_2a_ antibodies (Sigma) and anti-goat-HRP antibodies (Sigma) were used to detect the isotype of LACK-specific antibodies in sera. Reciprocal end-point titers of LACK specific IgG_1_ and IgG_2a_ antibodies were determined as with total IgG antibodies.

### Multiparameter flow cytometry

Spleens were collected from immunized animals two weeks after the last immunization (day 42). Single-cell suspensions were prepared in RPMI (Wisent) supplemented with 10% FBS (Wisent), 1 mM penicillin-streptomycin and 0.5 mM β-ME and 1×10^6^ splenocytes were plated in 96-well plates in a total of 100 µl. Cells were stimulated with purified LACK (20 µg/ml) and anti-CD28 (2 µg/ml) at 37°C for 2 hours. Brefeldin A (10 µg/ml, Sigma-Aldrich) was added and cells were incubated for 12 hours. Intra- and extracellular staining were performed according to IC staining protocol by eBioscience. In brief, FcγR was blocked and cells were stained for CD3 (Pe-Cy7), CD4 (FITC) and CD8 (PerCP-Cy5.5). Cells were permeabilized using Fixation/Permeabilization Buffer for 30 min and stained for IFN-γ (eFluor450), IL-2 (PE) and TNF-α (APC). Antibodies and reagents for intracellular staining were purchased from eBioscience. Samples were analyzed for intracellular cytokines using CyAn ADP flow cytometer (Beckman Coulter, Mississauga, ON) and data was analyzed using FlowJo software (Tree Star, Ashland, OR).

### *Ex vivo* stimulation of splenocytes and lymphocytes

Spleens or draining lymph nodes (popliteal and inguinal) were collected from immunized animals two weeks after the last immunization (day 42), 8 days after challenge (day 50) or at the end of the infection timeline. Single-cell suspensions were prepared in RPMI (Wisent) supplemented with 10% FBS (Wisent), 1 mM penicillin-streptomycin and 0.5 mM β-ME and 2×10^6^ cells were plated in 24-well plates in a total of 2 ml. Cells were stimulated with purified LACK (10 µg/ml) or soluble *Leishmania* antigen (SLA, 50 µg/ml) at 37°C under 5% CO_2_ for 3 days. Culture supernatants were collected and stored at −80°C until cytokine concentrations were quantified by ELISA. The concentration of IL-2, IL-4, IL-10, IFN-γ, and TNF-α were determined by ELISA Cytokine kits (eBiosicence) according to the manufacturer's protocol.

### Statistical analysis

Statistical significance between two groups was determined using the student T-test function of the StatView program, version 5.0 (SAS Institute Inc.; Cary, NC). *P*<0.05 was considered statistically significant.

## Results

### Expression of the *Leishmania major* LACK protein and biologically active mouse single chain IL-12 by *Lactococcus lactis*

To generate an effective live vaccine against *L. major*, we generated strains of *L. lactis* expressing the protective LACK antigen in three subcellular locations and a strain secreting mouse single chain IL-12 as adjuvant. In brief, we generated a strain of *L. lactis* expressing the LACK gene from *L. major* under the control of the nisin inducible promoter, leading to the expression of LACK in the cytoplasm (*L. lactis*/cytoLACK). To direct LACK expression outside of bacterial cells, we added the Usp45 secretion signal to its N-terminus (*L. lactis*/secLACK) [33], [34]. To attach LACK at the cell surface of *L. lactis*, the cell-wall anchoring domain of the M6 protein from *Streptococcus pyogenes* was added at the C-terminus of the secLACK construct (*L. lactis*/cwaLACK) [34]. Similarly to *L. lactis*/secLACK, a strain of *L. lactis* engineered to secrete mouse single chain IL-12 was generated (*L. lactis*/secIL-12). It was previously shown that the fusion of the two IL-12 subunits (p35–p40), creating single chain IL-12 (p70), increased the production of functional IL-12 when secreted from *L. lactis* [35].

**Figure 1:**
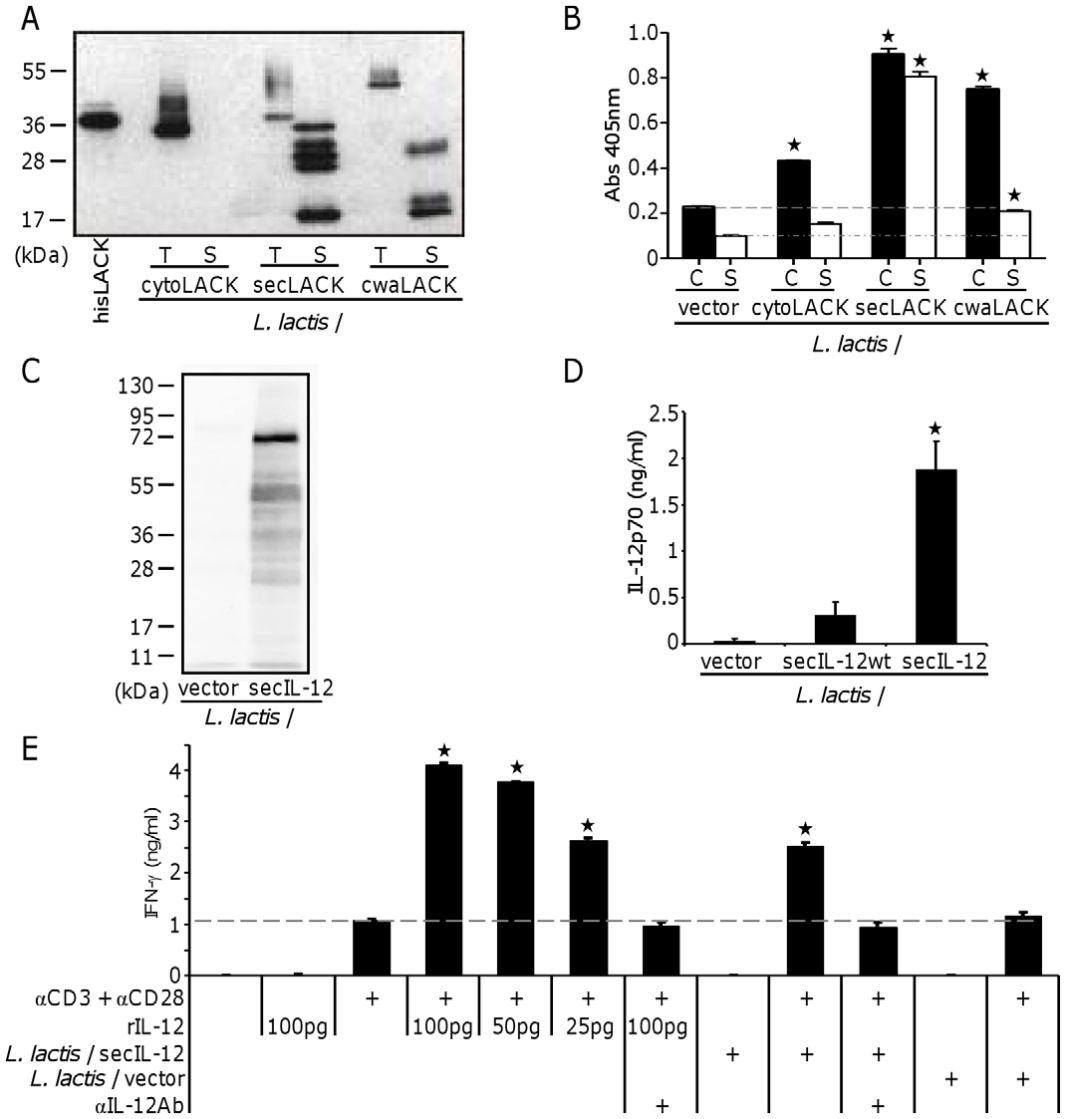
Expression and localization of the *Leishmania* LACK antigen and single-chain mouse IL-12 in *L. lactis*. (A) Western blot of total protein extracts of LACK-expressing strains of *L. lactis*. T, total cell extract; S, culture supernatant. (B) Localization of expressed LACK was confirmed by whole cell ELISA using anti-LACK antibody. C, intact cells; S, culture supernatant. * *P*<0.05 by unpaired T-test to *L. lactis*/vector. Data shown are representative of three independent experiments with similar results. (C) Total protein extracts of *L. lactis* were analyzed for IL-12 expression by Western blot using anti-IL-12p70 antibody. (D) Secretion of IL-12 by *L. lactis* was quantified using mouse IL-12p70 ELISA. * *P*<0.05 by unpaired T-test to *L. lactis*/secIL-12 wt. Data shown are representative of at least three independent experiments with similar results. (E) IFN-γ secretion by BALB/c splenocytes stimulated with αCD3/αCD28 in combination with rIL-12 or concentrated supernatant from *L. lactis* secreting IL-12 was determined by ELISA. * *P*<0.05 by unpaired T-test to αCD3/αCD28. Data shown are representative of three independent experiments with similar results.

Expression of LACK and IL-12 was confirmed by Western blot and ELISA. After inducing protein expression with nisin, total cell extracts and culture supernatants of LACK-expressing *L. lactis* strains were resolved on SDS-PAGE and blotted using LACK-specific antibodies (Fig. 1A). As expected, for *L. lactis*/cytoLACK we detected LACK only in the cell extract. For *L. lactis*/secLACK we were able to detect LACK in the bacterial supernatant as well as in the cell extract. The secLACK protein detected in the cytoplasm is larger than cytoLACK because it contains the secretion signal, which is cleaved during secretion. Finally, for *L. lactis*/cwaLACK we detected the LACK protein in both fractions. Since cwaLACK contains both the secretion signal and the cell wall anchoring domain, its size is larger than both cytoLACK and secLACK. The smaller bands detected in the culture supernatant possibly correspond to fragments of the LACK protein that were shed during renewal of the cell-wall. We further confirmed protein localisation by whole cell ELISA on live cells (Fig. 1B). Higher expression of LACK was detected at the cell surface of *L. lactis*/cytoLACK compared to background (*L. lactis*/vector), and it was not detected in the culture supernatant. As expected, LACK was detected at high levels on the cell surface and in the culture supernatant for *L. lactis*/secLACK whereas LACK expressed from *L. lactis*/cwaLACK was mainly detected at the cell surface. Detection of cwaLACK at the surface of intact cells demonstrates that the antigen protrudes sufficiently from the bacterial cell wall to be recognized by antibodies. The Western blot and whole cell ELISA results are coherent and confirm expression of the LACK antigen by *L. lactis* in different subcellular locations.

We also confirmed protein expression from *L. lactis*/secIL-12 after nisin induction. Whole cell protein extracts of *L. lactis*/vector and *L. lactis*/secIL-12 were resolved on SDS-PAGE and blotted using mouse IL-12-specific antibodies (Fig. 1C). Single chain mouse IL-12 was detectable from *L. lactis*/secIL-12 at approximately 70 kDa. To confirm protein secretion, we determined the concentration of secreted IL-12p70 in concentrated *L. lactis* culture supernatants by ELISA (Fig. 1D). To test if codon-optimization of the mouse single chain IL-12 gene led to an increase in protein production, we compared IL-12 concentration in supernatant from *L. lactis* strains carrying either the wild type (*L. lactis*/secIL-12 wt) [35] or the codon-optimized single chain mouse IL-12. In order to prevent degradation of IL-12 during secretion, *L. lactis* was grown in G3xM17. This medium possesses higher buffering capacity and suppresses the acid tolerance response of *L. lactis*, which leads to higher protein secretion [40]. We were able to detect secretion of mouse IL-12 by both strains. However, bacteria expressing the codon-optimized gene secreted approximately six times higher amounts of IL-12 giving a geometric mean of 1.886 ng/ml in codon-optimized compared to 0.316 ng/ml in wild-type. To determine whether the single chain IL-12 secreted by *L. lactis* was biologically active, splenocytes from BALB/c mice were stimulated with anti-CD3/anti-CD28 and concentrated supernatant from *L. lactis*/secIL-12 to assess its ability to stimulate IFN-γ production (Fig. 1E). Concentrated bacterial supernatant of the *L. lactis* strain expressing codon optimized single chain IL-12 was able to stimulate splenocytes to comparable levels as recombinant IL-12 (25 pg/ml). In both cases, addition of an anti-mouse IL-12 neutralizing antibody reduced IFN-γ secretion by splenocytes to background levels. These results confirm that mouse single chain IL-12 secreted by *L. lactis* is able to stimulate mouse splenocytes and hence is biologically active.

### Subcutaneous vaccination with cwaLACK- and IL-12-expressing *L. lactis* strains induces a significant delay in footpad swelling in *L. major* infected BALB/c mice

**Figure 2:**
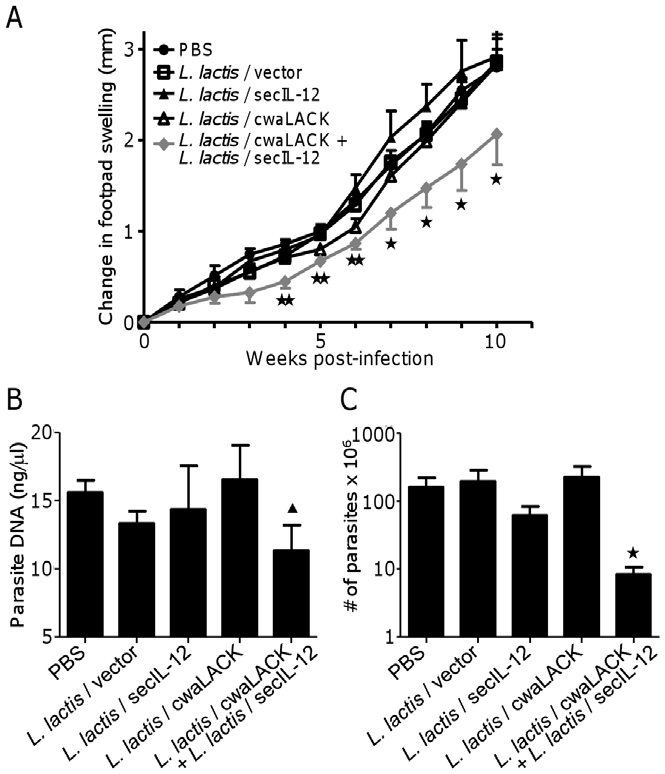
Immunization with *L. lactis*/cwaLACK and *L. lactis*/secIL-12 delays footpad swelling and reduces parasite burden in *L. major* infected BALB/c mice. Mice were immunized three times subcutaneously with PBS or different strains of *L. lactis* and challenged with *L. major* promastigotes into the right hind footpad. (A) Change in footpad swelling post-infection was determined by measuring the thickness of the infected footpad and subtracting the thickness of the contralateral, uninfected footpad at weekly intervals. Animals were sacrificed ten weeks after parasite challenge and parasite burden in infected footpads was determined by (B) PCR-ELISA or (C) limiting dilution. Mean and SEM of four to five mice per group are shown. * *P*<0.05; ** *P*<0.01 by unpaired T-test to PBS and *L. lactis*/vector. ^▴^ *P*<0.05 by unpaired T-test to PBS. Data shown are representative of three (A), one (B), or two (C) independent experiments with similar results.

The capacity of LACK- and IL-12-expressing *L. lactis* to protect mice against cutaneous *L. major* challenge was evaluated. BALB/c mice were subcutaneously immunized with one of the three LACK-expressing *L. lactis* strains in combination with *L. lactis* expressing secIL-12 or carrying the empty vector (total dose of 1×10^9^ live bacteria). As controls, mice were immunized with either PBS, *L. lactis*/vector or *L. lactis*/secIL-12 in combination with *L. lactis*/vector. Quantities of expressed proteins delivered per immunization were estimated by Western blot (*L. lactis*/cytoLACK: ∼14.8±0.9 µg, *L. lactis*/secLACK: ∼12.6±1.4 µg, *L. lactis*/cwaLACK: ∼8.7±0.7 µg, *L. lactis*/secIL-12: ∼5.3±0.1 µg). Mice were immunized every second week for a total of three immunizations. Two weeks after the final immunization, mice were challenged with 5×10^6^ stationary *L. major* promastigotes injected into the right hind footpad. Disease progression was monitored by measuring footpad swelling every week (Fig. 2A). Mice immunized with *L. lactis*/vector, *L. lactis*/secIL-12 or *L. lactis*/cwaLACK displayed a similar increase in footpad swelling as mice treated with PBS. However, animals immunized with *L. lactis* expressing cwaLACK in combination with *L. lactis* secreting IL-12 displayed a significant delay in footpad swelling compared to the groups immunized with PBS or *L. lactis*/vector, starting at four weeks post-infection. In contrast, animals immunized with *L. lactis*/cytoLACK or *L. lactis*/secLACK did not show reduced footpad swelling, when administered alone or in combination with *L. lactis*/secIL-12 (data not shown). The delay in footpad swelling in animals immunized with *L. lactis*/cwaLACK in combination with *L. lactis*/secIL-12 was associated with a significantly lower parasite burden in the footpad compared to controls as determined by PCR-ELISA and limiting dilution assay (Fig. 2B, 2C). Taken together, these findings demonstrate that mice immunized with *L. lactis*/cwaLACK and *L. lactis*/secIL-12 were partially protected against *L. major* challenge.

### Immunization with cwaLACK-expressing *L. lactis* induces antigen-specific humoral immune responses

**Figure 3:**
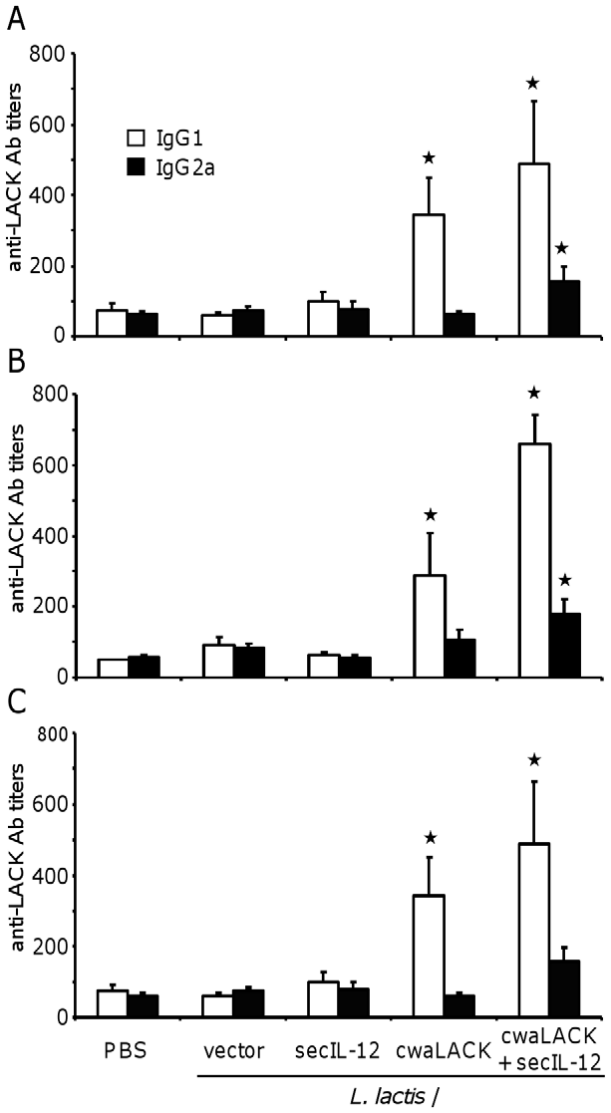
Antigen-specific humoral immune response following immunization with LACK-expressing *L. lactis*. Mice were immunized three times subcutaneously with PBS or different strains of *L. lactis* and blood was collected one week after the last immunization (A), four weeks after challenge (B) or ten weeks after challenge (C). LACK-specific IgG_1_ and IgG_2a_ antibody titers in sera were determined by ELISA. Mean and SEM of four to five mice per group are shown. * *P*<0.05 by unpaired T-test to PBS and *L. lactis*/vector. Data shown are representative of two independent experiments with similar results.

To characterize the immune response induced by the live vaccine, we analyzed antigen-specific humoral responses before parasite challenge (day 35, Fig. 3A) as well as at four (Fig. 3B) and ten weeks (Fig. 3C) after challenge. Animals immunized with *L. lactis*/cwaLACK and *L. lactis*/secIL-12 displayed mainly a T_H_2 humoral immune response against LACK characterized by antigen-specific IgG_1_ titers. Additionally, LACK-specific IgG_2a_ antibodies were detected in these animals before and after challenge, indicating the induction of a weak T_H_1 immune response. In contrast, animals immunized with only *L. lactis*/cwaLACK, showed LACK-specific IgG_1_ titers but antigen-specific IgG_2a_ titers were not detectable, indicating an antigen-specific T_H_2 response. Generally, LACK-specific IgG_1_ and IgG_2a_ antibody titers in immunized animals were low, suggesting that the live vaccine induced weak humoral immune responses.

### Induction of antigen-specific multifunctional T_H_1 cells correlates with protection against *L. major* infection

**Figure 4:**
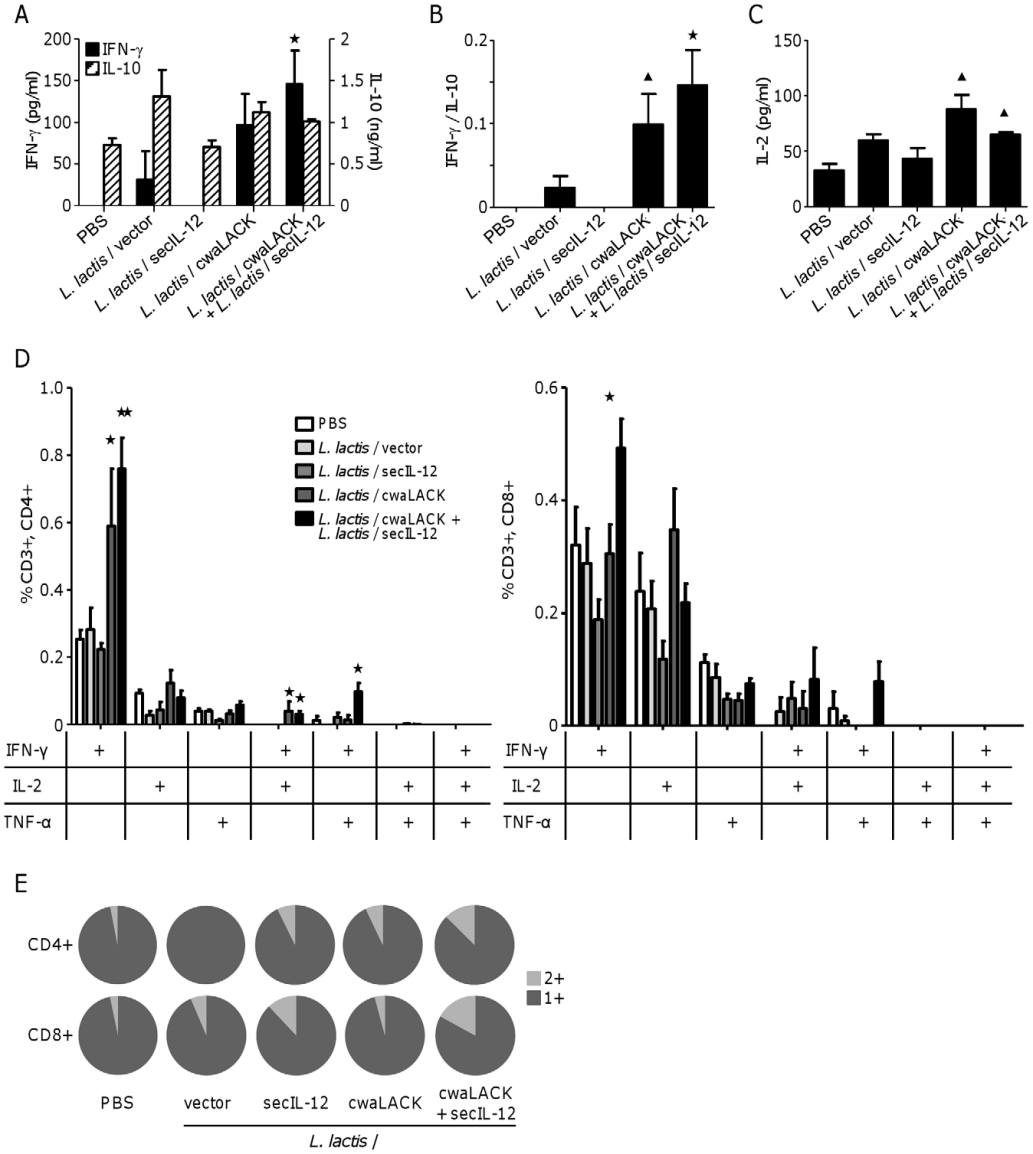
Immunization with *L. lactis*/cwaLACK and *L. lactis*/secIL-12 induces systemic T_H_1 immune responses and multifunctional T_H_1 cells pre-challenge. Mice were immunized three times subcutaneously with PBS or different strains of *L. lactis*. Mice were sacrificed two weeks after the last immunization and splenocytes were restimulated with purified LACK antigen *ex vivo*. (A, B) IFN-γ, IL-10, and (C) IL-2 secretion after three days of restimulation was determined by ELISA. (D) Splenocytes were restimulated for 16 hours and cytokine expression was analyzed by flow cytometry. Frequencies of CD4^+^ and CD8^+^ T cells positive for IFN-γ, IL-2, or TNF-α or the combination of the different cytokines are shown. (E) Fraction of the total CD4^+^ or CD8^+^ response comprising cells expressing any two cytokines (2+), or any one cytokine (1+). Mean and SEM of four to five mice per group are shown. * *P*<0.05; ** *P*<0.01 by unpaired T-test to PBS and *L. lactis*/vector. ^▴^ *P*<0.05 by unpaired T-test to PBS. Data shown are representative of two independent experiments with similar results.

To evaluate the cellular immune responses induced by the live vaccine, we assessed cytokine secretion in response to LACK antigen by splenocytes from immunized animals. Mice were immunized as described above and sacrificed two weeks after the last immunization. Splenocytes of animals immunized with *L. lactis*/cwaLACK and *L. lactis*/secIL-12 secreted significantly higher amounts of IFN-γ upon antigen restimulation compared to animals immunized with PBS or *L. lactis*/vector (Fig. 4A). Since the ratio of IFN-γ to IL-10 is a correlate of immune protection against *L. major* [41], [42], we also quantified IL-10 secretion by restimulated splenocytes. Animals immunized with *L. lactis*/cwaLACK and *L. lactis*/secIL-12 displayed a significant increase in the IFN-γ/IL-10 ratio compared to both PBS and *L. lactis*/vector control groups (Fig. 4A, 4B). In addition, restimulated splenocytes from animals immunized with *L. lactis*/cwaLACK or *L. lactis*/cwaLACK and *L. lactis*/secIL-12 secreted significantly higher amounts of IL-2 upon antigen recall, compared to animals immunized with PBS (Fig. 4C). Secretion of the T_H_2 cytokine, IL-4, was not detectable from splenocytes of any of the groups after LACK restimulation (data not shown).

To further characterize the cell-mediated immune responses induced by the live vaccine, we determined T_H_1 cytokine production by splenic CD4^+^ and CD8^+^ T cells using multiparameter flow cytometry. Splenocytes of immunized mice were cultivated *in vitro* with or without LACK and stained for CD3, CD4, CD8, IFN-γ, TNF-α, and IL-2 expression. Cells were gated on live CD3^+^, separated into CD4^+^ and CD8^+^ cells, and divided in seven distinct populations of cytokine-producing cells. The quality of the T_H_1 immune response induced by a vaccine is based on the frequency of these distinct populations and there is evidence that multifunctional T_H_1 cells are mediators of protection against *L. major* challenge [41]. Significantly higher numbers of IFN-γ secreting CD4^+^ T cells were found in restimulated splenocytes from animals immunized with *L. lactis*/cwaLACK or *L. lactis*/cwaLACK and *L. lactis*/secIL-12 compared to control groups (Fig. 4D). Furthermore, immunization with the same *L. lactis* strains induced multifunctional, antigen-specific CD4^+^ T cells. Higher frequency of CD4^+^ T cells secreting IFN-γ and IL-2, and CD4^+^ T cells producing IFN-γ and TNF-α were detected in splenocytes from these groups upon antigen recall (Fig. 4E). Immunization with *L. lactis*/cwaLACK and *L. lactis*/secIL-12 induced significantly higher numbers of IFN-γ^+^ CD8^+^ T cells. The frequency of multifunctional CD8^+^, IFN-γ^+^ and IL-2^+^ and multifunctional CD8^+^, IFN-γ^+^, TNF-α^+^ T cells, was also increased but not significantly compared to controls.

Taken together, these results suggest that protection against *L. major* infection in animals immunized with *L. lactis*/cwaLACK and *L. lactis*/secIL-12 correlates with a systemic antigen-specific T_H_1 immune response and the induction of LACK-specific mono- and multifunctional T_H_1 cells before challenge.

### Protection against *L. major* infection correlates with *Leishmania*-specific T_H_1 immune response after parasite challenge

**Figure 5:**
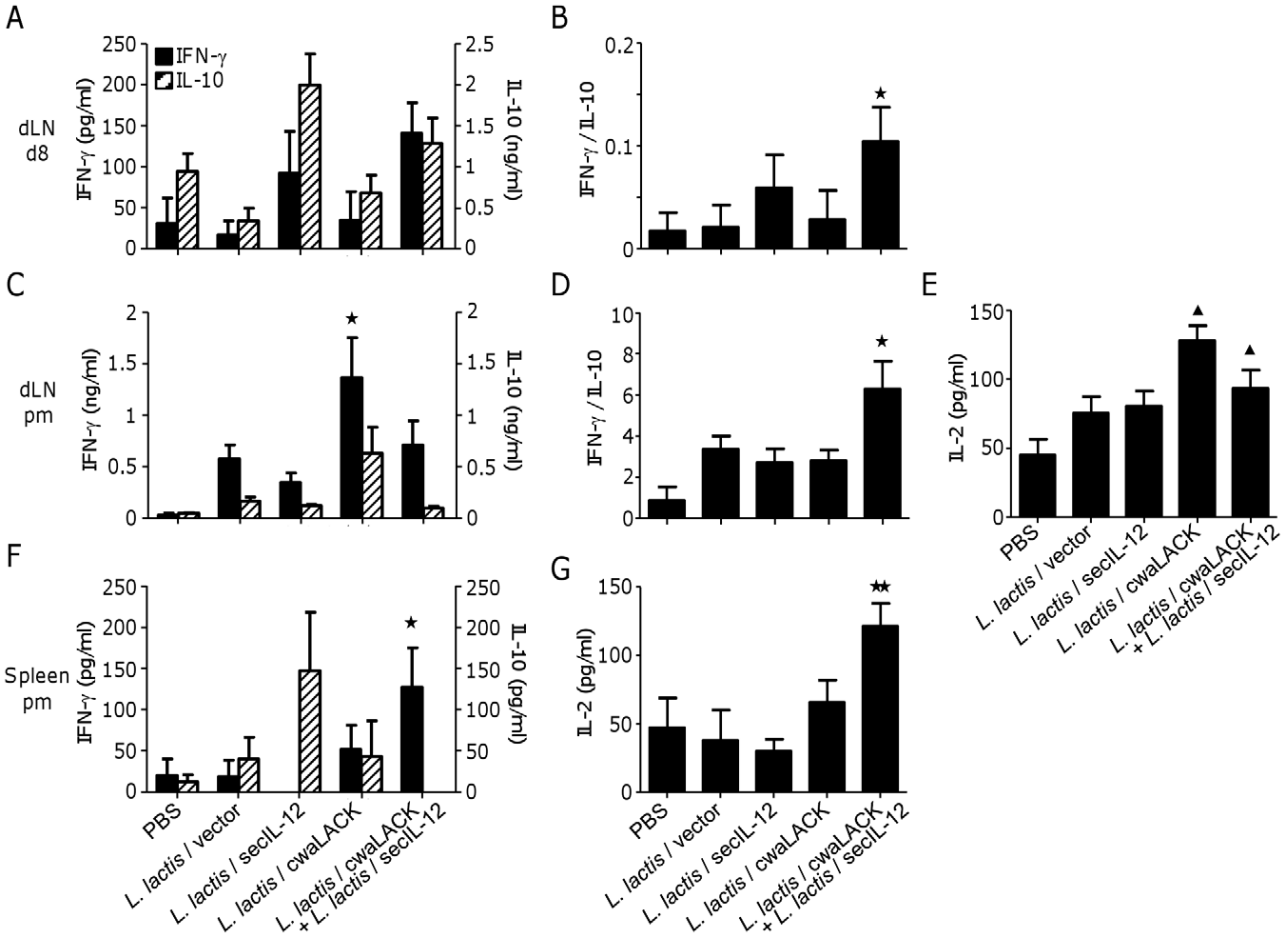
Protection against *L. major* infection correlates with *Leishmania*-specific T_H_1 response in immunized animals. Mice were immunized three times subcutaneously with PBS or different strains of *L. lactis* and challenged with *L. major*. Mice were sacrificed eight days (A, B), or ten weeks (C–G) after challenge (pm: *post mortem*). Lymph node cells (A–E), or splenocytes (F, G) were restimulated with SLA *ex vivo*. IFN-γ, IL-10, and IL-2 secretion by restimulated draining lymph node cells and splenocytes were determined by ELISA. Mean and SEM of four to five mice per group are shown. * *P*<0.05; ** *P*<0.01 by unpaired T-test to PBS and *L. lactis*/vector. ^▴^ *P*<0.05 by unpaired T-test to PBS. Data shown are representative of two independent experiments with similar results.

To examine the nature of protection against *L. major* induced by *L. lactis*/cwaLACK and *L. lactis*/secIL-12, cytokine responses in lymph node cells and splenocytes of immunized animals were analyzed eight days and ten weeks after parasite challenge (Fig. 5).

Draining lymph nodes were collected and restimulated with soluble *Leishmania* antigen (SLA) and cytokine secretion was quantified (Fig. 5A–E). We first determined the ratio of IFN-γ to IL-10 secretion by restimulated lymphocytes at day 8 and found that only animals immunized with *L. lactis*/cwaLACK and *L. lactis*/secIL-12 displayed a significantly higher ratio compared to animals immunized with PBS or *L. lactis*/vector (Fig. 5A, 5B). We did not detect significant differences in IL-2 or IL-4 production from restimulated lymph node cells among the groups (data not shown). As expected, no antigen-specific cytokine secretion was detected in restimulated splenocytes eight days after parasite challenge (data not shown). At ten weeks post-challenge, the IFN-γ/IL-10 ratio was significantly higher in restimulated lymph node cells from animals immunized with *L. lactis*/cwaLACK and *L. lactis*/secIL-12 compared to controls (Fig. 5C, 5D). We also found elevated IL-2 secretion by restimulated lymph node cells of animals immunized with live bacteria but significantly higher amounts in animals immunized with *L. lactis*/cwaLACK or *L. lactis*/cwaLACK and *L. lactis*/secIL-12 compared to PBS (Fig. 5E). IL-4 secretion by restimulated lymphocytes was detectable but no statistically significant differences were found between the groups (data not shown).

Similar results were obtained in SLA-restimulated splenocytes in animals ten weeks after parasite challenge (Fig. 5F, 5G). Animals immunized with *L. lactis*/cwaLACK and *L. lactis*/secIL-12 displayed significantly higher secretion of IFN-γ compared to animals immunized with PBS and *L. lactis*/vector (Fig. 5F). However, the IFN-γ/IL-10 ratio could not be calculated since IL-10 secretion by splenocytes of animals immunized with *L. lactis*/cwaLACK and *L. lactis*/secIL-12 was not detectable (Fig. 5F). Furthermore, IL-2 secretion by SLA-restimulated splenocytes from animals immunized with *L. lactis*/cwaLACK and *L. lactis*/secIL-12 was significantly higher compared to all other groups (Fig. 5G). IL-4 secretion was not detectable in any of the groups (data not shown).

In summary, these results indicate that animals immunized with *L. lactis*/cwaLACK and *L. lactis*/secIL-12 display a strong anti-*Leishmania* T_H_1 immune response detectable in draining lymph nodes shortly after parasite infection as well as locally and systemically at 10 weeks post-challenge.

## Discussion

Leishmaniasis presently affects over 12 million individuals worldwide and approximately 2 million new cases occur every year. Current treatments are labour-intensive, expensive, cause severe side effects and emerging drug resistance has been reported [1], [2]. Hence, further interventions against the disease are needed. Vaccination is the most cost-effective means to control infectious disease. Since leishmaniasis is mainly a disease of the developing world, a vaccine against *Leishmania* has to be affordable to the population in need, preferably stable at room temperature and easy to administer, as well as safe and effective. The use of *L. lactis* as a live vaccine shows promising results against various diseases and has recently been shown to be safe for oral administration in humans [26]. Furthermore, a live vaccine using strains of *L. lactis* would be inexpensive to produce, stored simply as lyophilised pellets and could be easily administered via mucosal routes. As a first step towards the generation of *L. lactis* live vaccine against *Leishmania*, we report effective subcutaneous immunization against *L. major* infection in the mouse model.

We generated strains of *L. lactis* expressing the protective *Leishmania* antigen LACK in three different subcellular locations and a strain of *L. lactis* secreting biologically active mouse IL-12. We demonstrated that *L. lactis* expressing codon-optimized IL-12 secretes approximately six times more IL-12 than the previously described strain expressing the murine wild-type gene [35]. Subcutaneous immunization using these strains was well tolerated by animals. Formation of skin ulcerations was occasionally detected at sites of injection, however they resolved during the following weeks. Only immunization with *L. lactis*/cwaLACK in combination with *L. lactis*/secIL-12 conferred a delay in footpad swelling and significant reduction in parasite burden in *L. major* infected BALB/c mice. It is well established that the immunogenicity of an antigen expressed by *L. lactis* depends on its subcellular location [24]. However, it is difficult to predict the most immunogenic expression strategy, since this depends on both the antigen expressed and on the route of immunization. Our result showing that only LACK anchored to the bacterial cell-wall was able to induce a protective immune response against *L. major* challenge is in accordance with the finding of Norton *et al*. [43]. They showed that subcutaneous immunization with *L. lactis* expressing tetanus toxin fragment C (TTFC) anchored to the cell-wall was more immunogenic than immunization with bacteria expressing the antigen in the cytoplasm or secreted, and was able to provide better protection against lethal challenge. Additionally, our group previously showed that subcutaneous immunization with *L. lactis* expressing A2 anchored to the cell-wall induced the highest level of antigen-specific antibody titers compared to other expression strategies and reduced parasite burdens in *L. donovani*-challenged animals [29]. Therefore, we hypothesized that for subcutaneous immunization with *L. lactis*, the expression of the antigen anchored to the cell-wall would be the most immunogenic. We showed in this study that immunization with *L. lactis*/cwaLACK alone failed to induce a protective immune response against *L. major* challenge, indicating the necessity for co-administration of *L. lactis*/secIL-12 as a T_H_1-inducing adjuvant. This is similar to previous studies where recombinant LACK was shown to induce protection against parasite challenge only if the antigen was administered with T_H_1-inducing agents or as a DNA vaccine (reviewed in [7]). This also holds true for immunization using LACK-expressing strains of bacteria. Lange *et al.* reported that intravenous immunization with *Salmonella* expressing LACK did induce *Leishmania*-specific T_H_1 immune response in BALB/c mice but did not provide protection against parasite challenge [17]. However, they reported successful immunization against *L. major* challenge by heterologous prime-boost immunization using LACK DNA and LACK-expressing *Salmonella enterica*. Soussi *et al.* showed that intravenous immunization using LACK-expressing *Listeria monocytogenes* failed to provide protection against *L. major* challenge in BALB/c mice despite the induction of a LACK-specific T_H_1 immune response [15]. However, the same group reported partial protection against *L. major* infection if animals were immunized intraperitoneally or intragastrically with LACK-expressing *Listeria* [16]. With our *L. lactis* live vaccine, we obtained protection comparable to the *Salmonella* prime-boost strategy but better protection compared to immunization using *Listeria*. However, the use of attenuated pathogenic bacteria as live vaccine raises safety concerns, which is not an issue when using a non-pathogenic and food grade bacterium such as *L. lactis*.

Another purpose of this study was to determine the mechanisms of action of our *L. lactis* live vaccine. We first assessed the humoral immune response induced after immunization. We found that immunization with *L. lactis*/cwaLACK induced LACK-specific IgG_1_ antibodies, indicating an antigen-specific T_H_2 immune response. However, in animals immunized with *L. lactis*/cwaLACK and *L. lactis*/secIL-12, LACK-specific IgG_2a_ was also detectable in sera pre- and post-challenge, indicating the induction of a mixed T_H_1/T_H_2 response. In general, antibody titers in immunized animals were low, indicating a weak humoral response. This is in contrast to the strong humoral responses induced by subcutaneous immunization using *L. lactis* expressing the *Leishmania* antigen A2 or expressing TTFC anchored to the bacteria cell wall [29], [43]. The differences in antibody titers could be explained by lower expression of the LACK antigen, or differences in the processing of the antigens by immune cells. However, induction of *Leishmania*-specific IgG has been shown to exacerbate disease in *L. major*-infected mice [44], [45]. Thus, the induction of a weak humoral immune response might contribute to the protective efficacy of our vaccine against *L. major* challenge.

To further characterize the *L. lactis* live vaccine, we assessed the cell-mediated immune response induced before and after *L. major* challenge. Immunization with *L. lactis*/cwaLACK and *L. lactis*/secIL-12 induced an antigen-specific T_H_1 response. Only animals immunized with this combination of the *L. lactis* strains showed a significant increase in the IFN-γ/IL-10 ratio, which is an important correlate of immune protection against *L. major* [42]. In addition, immunization with *L. lactis*/cwaLACK and *L. lactis*/secIL-12 induced antigen-specific multifunctional T_H_1 cells. Induction of these cells, which are capable of producing several T_H_1 cytokines, was correlated with protection against *L. major* infection [41]. Immunization using *L. lactis*/cwaLACK alone also induced antigen specific CD4^+^, IFN-γ^+^ T cells albeit at a lower level. Similarly, fewer LACK-specific multifunctional CD4^+^ and CD8^+^ cells expressing IFN-γ and TNF-α were detected in these animals. This indicates that immunization with *L. lactis*/cwaLACK is able to induce IFN-γ production and multifunctional T_H_1 cells. However, the degree and/or the quality of the T_H_1 immune response induced by *L. lactis*/cwaLACK was not sufficient to protect against *L. major* challenge and a protective immune response was only achieved with the adjuvant effect of *L. lactis*/secIL-12.

Protection against *L. major* infection correlated with a strong *Leishmania*-specific T_H_1 immune response post-challenge in immunized animals. This was detectable as early as eight days, but also at ten weeks after challenge, in lymph nodes draining the site of infection. Similarly, a strong systemic T_H_1 response was detected at ten weeks after immunization, indicated by the high levels of IFN-γ and the absence of IL-10 in restimulated splenocytes. Animals immunized with only *L. lactis*/cwaLACK did not display a *Leishmania*-specific T_H_1 immune response, indicating the need for *L. lactis*/secIL-12 as adjuvant to induce a protective immune response against the parasite. These observations show that in our infection model, vaccine efficacy can be predicted by the ratio of antigen-specific production of IFN-γ to IL-10 and that protected animals continue to display a *Leishmania*-specific T_H_1 immune response. Furthermore, the ratio of IFN-γ to IL-10 was also an indicator of the protective immune response post-challenge, which is in accordance to findings in other *L. major* infection models [42], [46].

In summary, we showed that the LACK antigen of *Leishmania*, as well as mouse IL-12, can be effectively expressed by *L. lactis*. We further demonstrated that subcutaneous immunization with *L. lactis*/cwaLACK and *L. lactis*/secIL-12 delayed footpad swelling and significantly reduced parasite burden in *L. major* infected BALB/c mice. Protection correlated with the induction of LACK-specific multifunctional T_H_1 cells before challenge and *Leishmania*-specific T_H_1 immune response after challenge. The *L. lactis* strains generated provide the basis for the development of an inexpensive, safe and effective *Leishmania* vaccine.
